# Editorial: Benefits of bioactive plant-based compounds supplementation on cardiovascular and metabolic health

**DOI:** 10.3389/fnut.2023.1197450

**Published:** 2023-04-28

**Authors:** Azizah Ugusman, Chua Kien Hui, Wan Amir Nizam Wan Ahmad

**Affiliations:** ^1^Department of Physiology, Faculty of Medicine, Universiti Kebangsaan Malaysia, Kuala Lumpur, Malaysia; ^2^Biomedicine Programme, School of Health Sciences, Universiti Sains Malaysia, Kubang Kerian, Malaysia

**Keywords:** plant-based supplements, bioactive compound, cardiovascular diseases, metabolic syndrome, antioxidant

The aim of this Research Topic was to highlight research that utilize bioactive plant-based compounds as an intervention strategy for cardiovascular and metabolic health in both preclinical and clinical phases. This Research Topic compiles eight articles, including three reviews and five original research articles from prominent scientists in the field. The content of each article is summarized below.

Plant-based products such as honey have been investigated for their potential effect on obesity. Hence, Ugusman et al. systematically reviewed the recent literature concerning the effects of honey on obesity in preclinical and clinical settings. Honey exerts anti-obesity effects in animal studies by reducing body weight, body mass index, body fat composition, adiposity index, adipocyte hypertrophy and adipocyte hyperplasia. However, most clinical trials show insignificant results due to the small sample size, limited treatment duration and the presence of confounding factors such as diet and physical activity.

Carotenoids are a group of antioxidants naturally present in plants. Zhu et al. investigated the associations of serum carotenoid (a-carotene, b-cryptoxanthin, lutein/zeaxanthin, trans-lycopene, *trans*-b-carotene, and *cis*-b-carotene) concentrations with the prevalence of hypertension in the general adult population using data from National Health and Nutrition Examination Survey 2001–2006. Their findings indicate that higher levels of all six mixed serum carotenoids are correlated with decreased prevalence of hypertension, among which b-carotene exerts the most significant effect, which may provide a basis and direction for further studies.

Lutein is useful in lowering blood pressure and preventing age-related degenerative diseases. Human obtains lutein solely from their diet, especially from green leafy vegetables. A randomized controlled trial by Xiong et al. investigated the dose-response relationship between oral lutein intake and plasma lutein concentration in a young Chinese population. The results showed that a single oral dose of 40 mg of lutein more significantly improved plasma lutein concentration than a single oral dose of 10 and 20 mg of lutein. This dose-response relation was more pronounced among men. Hence, oral lutein intake is an applicable method to raise plasma lutein concentration.

Non-alcoholic fatty liver disease (NAFLD) is a chronic and progressive metabolic disease characterized by excessive fat deposition in the hepatocytes in the absence of alcohol exposure or other identifiable causes. A systematic review and meta-analysis by Wang et al. evaluated the adjuvant therapy effects of various nutritional interventions which include antioxidants, probiotic/symbiotic/prebiotic, fatty acid supplements, vitamin D, and whole grain on NAFLD patients. The study suggests that antioxidant and probiotic/symbiotic/prebiotic supplements alone or in combination with other therapies may be a promising regimen for NAFLD patients.

Dong and Yang reported that a higher dietary fiber intake is associated with a lower prevalence of stroke and non-fatal myocardial infarction in adults in the United States. The demographic, socioeconomic and disease status do not affect these association. Hence, increase in dietary fiber intake could confer protection against cardiovascular and cerebrovascular diseases. Meanwhile, a systematic review and meta-analysis by Yang et al. evaluated the effect of omega-3 fatty acids (OM3-FA) or their combination with statins on the lipid profile in patients with hypertriglyceridemia. They found that the use of OM3-FA either as monotherapy or in combination with statins may potentially reduce the levels of triglycerides, total cholesterol, very low-density lipoprotein cholesterol, non-high-density lipoprotein cholesterol (non-HDL-C), apolipoprotein (Apo)-B and Apo-AI, while increasing the levels of low-density lipoprotein cholesterol and HDL-C.

Oxidative stress has been implicated in cardiovascular and renal complications of Fabry disease (FD). *Camellia sinensis* or green tea is high in epigallocatechin-3-gallate, a catechin that is a potent antioxidant. A pilot study by Bertoldi et al. demonstrated that early additive green tea treatment reduced oxidative stress and alleviated cardio and cerebrovascular-renal complications related to oxidative stress in FD. This study highlights the fundamental importance of early treatment for FD and the positive effect of an adjuvant antioxidant therapy toward reducing oxidative stress-associated complications.

Lycopene is a carotenoid that is abundantly present in red fruits and vegetables. Zhong et al. studied the effects of dietary lycopene intake on cardiovascular mortality among chronic kidney disease (CKD) patients. The authors demonstrated that the concentration of lycopene tended to decline with increasing age in CKD patients. Additionally, high concentrations of serum lycopene could significantly reduce all-cause mortality and cardiovascular mortality in CKD patients. Therefore, maintaining serum lycopene concentrations in CKD patients could lower their mortality risk.

## Author contributions

AU took the initiative to draft the manuscript. CH and WW contributed to it. All authors approved the final draft.

